# Development of a novel automatic ascites filtration and concentration equipment with multi‐ring‐type roller pump units for cell‐free and concentrated ascites reinfusion therapy

**DOI:** 10.1111/aor.13681

**Published:** 2020-06-02

**Authors:** Toshiya Okahisa, Masahiro Sogabe, Tadahiko Nakagawa, Kumiko Tanaka, Tetsu Tomonari, Tatsuya Taniguchi, Akira Takahashi, Yohsuke Kinouchi, Junji Nishioka, Naoki Igata, Hiroaki Yanagawa, Takatoshi Komatsu, Yoshiaki Ohnishi, Masashi Fukuhara, Masashi Ishikawa, Hiroshi Shibata, Hirohiko Shinomiya, Masahiko Nakasono, Fumiko Kishi, Keiko Komai, Yayoi Tatsuki, Toru Murashima, Yoshihiro Deguchi, Hiroshi Aramaki, Hideyuki Fukumitsu, Tetsuji Takayama

**Affiliations:** ^1^ Department of General Medicine and Community Health Science, Institute of Biomedical Sciences Tokushima University Graduate School Tokushima Japan; ^2^ Department of Health and Nutrition, Nursing Dietetics Department The University of Shimane Izumo Japan; ^3^ Department of Gastroenterology and Oncology, Institute of Biomedical Sciences Tokushima University Graduate School Tokushima Japan; ^4^ Department of Preventive Environment and Nutrition, Institute of Biomedical Sciences Tokushima University Graduate School Tokushima Japan; ^5^ Department of Electrical and Electronic Engineering Institute of Socio Techno Sciences Tokushima University Graduate School Tokushima Japan; ^6^ Course of Medical Science, Graduate School of Medical Sciences Tokushima University Graduate School Tokushima Japan; ^7^ Faculty of Medicine, Student Lab Tokushima University Tokushima Japan; ^8^ Clinical Trial Center for Developmental Therapeutics Tokushima University Hospital Tokushima Japan; ^9^ Department of Clinical Engineering, Division of Clinical Technology Tokushima University Hospital Tokushima Japan; ^10^ Dialysis Center Shikoku Central Hospital of the Mutual Aid Association of Public School Teachers Shikokuchuo Japan; ^11^ Department of Gastroenterology Tokushima Prefectural Central Hospital Tokushima Japan; ^12^ Department of Gastroenterology Yoshinogawa Medical Center Yoshinogawa Japan; ^13^ Department of Internal Medicine Tsurugi Municipal Handa Hospital Tsurugi Japan; ^14^ Department of Internal Medicine Tokushima Municipal Hospital Tokushima Japan; ^15^ Medical Device Business Division Takatori Corporation Kashihara Japan

**Keywords:** automatic ascites processing, cell‐free and concentrated ascites reinfusion therapy, refractory ascites

## Abstract

Cell‐free and concentrated ascites reinfusion therapy (CART) is an effective therapy for refractory ascites. However, CART is difficult to perform as ascites filtration and concentration is a complicated procedure. Moreover, the procedure requires the constant assistance of a clinical engineer or/and the use of an expensive equipment for the multi‐purpose blood processing. Therefore, we developed a CART specialized equipment (mobility CART [M‐CART]) that could be used safely with various safety measures and automatic functions such as automatic washing of clogged filtration filter and self‐regulation of the concentration ratio. Downsizing, lightning of the weight, and automatic processing in M‐CART required the use of newly developed multi‐ring‐type roller pump units. This equipment was approved under Japanese regulations in 2018. In performing 41 sessions of CART (for malignant ascites, 22 sessions; and hepatic ascites, 19 sessions) using this equipment in 17 patients, no serious adverse event occurred. An average of 4494 g of ascites was collected and the total amount of ascites was processed in all the sessions without any trouble. The mean weight of the processed ascites was 560 g and the mean concentration ratio was 8.0. The ascites were processed at a flow rate of 50 mL/min. The mean ascites processing time was 112.5 minutes and a 106.5‐minutes (95.2%) ascites processing was performed automatically. The operator responded to alarms or support information 3.2 times on average (3.1 minutes, 2.1% of ascites processing time). Human errors related to ascites processing were detected by M‐CART at 0.4 times per session on average and were appropriately addressed by the operator. The frequencies of automatic washing of clogged filtration filter and self‐regulation of the concentration ratio were 31.7% and 53.7%, respectively. The mean recovery rates (recovery dose) of protein, albumin, and immunoglobulin G were 72.9%, 72.9%, and 71.2% (65.9 g, 34.9 g, and 13.2 g), respectively. Steroids were administered in 92.7% of the sessions to prevent fever and the mean increase in body temperature was 0.53°C. M‐CART is a compact and lightweight automatic CART specialized equipment that can safely and easily process a large quantity of ascites without the constant assistance of an operator.

## 1 INTRODUCTION

Cell‐free and concentrated ascites reinfusion therapy (CART) is the treatment for refractory ascites, which resulted from a disease such as advanced cancer or liver cirrhosis.^1^, ^2^, ^3^ CART was approved in Japan in 1981 by the National Health Insurance and was initially performed in patients with hepatic ascites caused by liver cirrhosis.^4^, ^5^ Recently, CART has been widely performed in patients with malignant ascites and more than 85% of patients who underwent CART had malignant ascites as reported in the post‐marketing surveillance.^6^ CART comprises three processes: (a) drainage process, which involves the collection of ascites by paracentesis; (b) filtration and concentration process, which involves the complete removal of cell components and bacteria using a filtration filter and removal of superabundant water using a concentration filter; and (c) treated ascitic intravenous drip infusion process. CART is effective for the relief of symptoms and prevents the worsening of nutritional status through the drainage of ascites.^6^, ^7^, ^8^, ^9^ Furthermore, the use of a combination of chemotherapy and CART^10^ and the application of sampling cells to cancer vaccines^11^ were reported, and CART was recognized as an effective treatment that can support cancer therapy.

The ascites processing in CART had been performed using a drop‐type method,^12^ an aspiration‐type method (KM‐CART),^13^ a pump‐type method using the roller pump equipment without the mode for CART,^14^, ^15^ a combination of these methods (eg, DC‐CART),^16^ and a pump‐type method using the expensive multi‐purpose blood processing equipment with the mode for CART.^5^ At the priming phase, the filtration filter must be checked for any leaks to prevent the contamination of cancer cells or bacteria by the corruption of the hollow fiber.^17^ Using the multi‐purpose blood processing equipment with the mode for CART, this leak check is automatically carried out, but is performed in other methods manually. When the filtration filter is clogged, reduction of filtration flow, drainage of the clogging substances in the hollow fibers, or manual washing of filter with normal saline using a syringe was performed.^13^ However, clogging of the filtration or concentration filters sometimes prevented the processing of the entire volume of collected ascites.^6^, ^15^

Therefore, we developed a novel CART specialized equipment that could process a large quantity of ascites safely and automatically without requiring the constant assistance of an operator by improving the automatic functions and safety measures. In this study, we aimed to report the course of the development, structure, and characteristics of the developed equipment; usability at priming; and the clinical evaluations that involved the performance of CART in 41 sessions.

## 2 MATERIALS AND METHODS

### 2.1 Development project

We built a consortium consisting of Tokushima University, Tokushima University Hospital, a medium‐sized manufacturing company (Takatori Corporation, Nara, Japan), and affiliated hospitals to develop a novel CART specialized equipment and established a research and development room in the incubation center (Fujii Memorial Institute of Medical Sciences) inside the Tokushima University Hospital premises. Initially, we developed a CART specialized equipment (tractable CART [T‐CART]), which could be used easily, while being adopted by the Program to Support Development of Medical Equipment and Devices to Solve Unmet Medical Needs (Grant No. 25‐017, fiscal year 2013, Ministry of Economy, Trade and Industry [METI]) and Development of Medical Device through Collaboration between Medicine and Industry (Grant No. 25‐017, fiscal year 2014‐2015, Japan Agency for Medical Research and Development [AMED]). Through the accompanying consultation of these projects, we received continuous advice on commercialization by experts from various fields.

**FIGURE 1:**
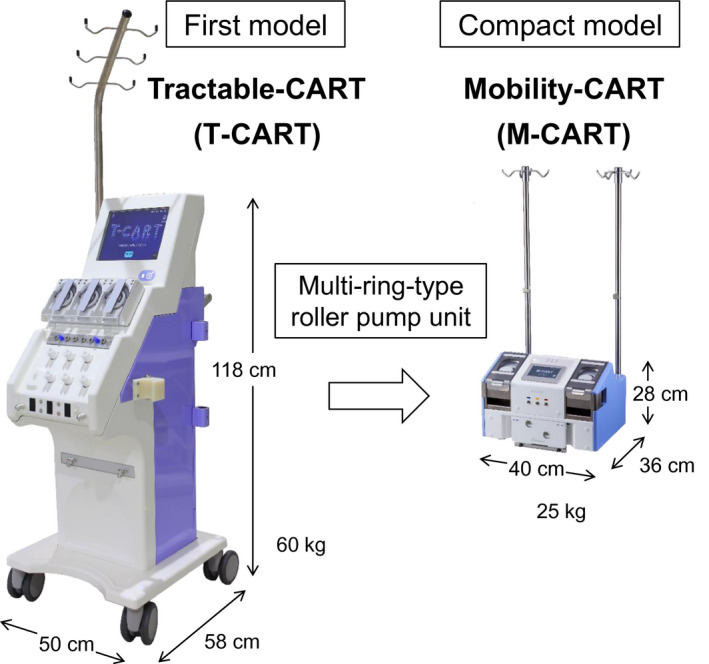
Developed CART specialized equipment. T‐CART was improved by introducing the multi‐ring‐type roller pump unit and a compact CART specialized equipment (M‐CART) was completed. In M‐CART, 70% downsizing and 60% lightning of the weight were achieved [Color figure can be viewed at wileyonlinelibrary.com]

During the course of the development, the introduction of the design thinking using the Dialogue Tools for Innovation (Seeds and Needs Creation Reinforcement Support Project in Universities, fiscal year 2014, Ministry of Education, Culture, Sports, Science and Technology) was connected to a novel idea to perform all automatic processes, from priming to the filtration and concentration, using only one pump unit and led to the development of the multi‐ring‐type roller pump unit (Mediation Research and Development Promotion Business to Backbone, the Small and Medium‐sized Enterprise, Grant No. 27J1130, fiscal year 2015‐2016, New Energy and Industrial Technology Development Organization [NEDO]). T‐CART was improved after the introduction of this pump unit and a downsized lightweight low price model (mobility CART [M‐CART]) was completed (Figure 1). M‐CART was approved under Japanese regulations in March 2018 and was released in December 2018.

### 2.2 Multi‐ring‐type roller pump unit

**FIGURE 2:**
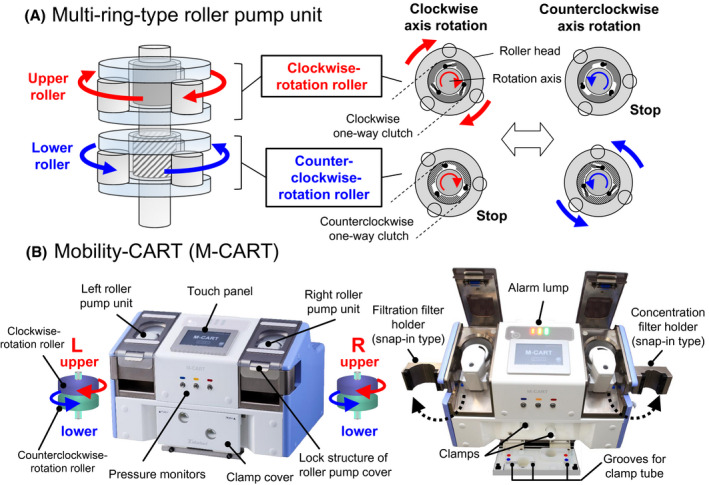
Structure and motions of the multi‐ring‐type roller pump unit (A) and equipment of M‐CART (B). The multi‐ring‐type roller pump unit is composed of roller heads each equipped with a one‐way clutch. Roller heads turn according to the direction of the rotation and the roller head that does not turn is referred to as the clamp. M‐CART is equipped with two multi‐ring‐type roller pump units. Each pump is equipped with an upper clockwise‐rotation roller head and a lower counterclockwise‐rotation roller head [Color figure can be viewed at wileyonlinelibrary.com]

The multi‐ring‐type roller pump unit has more than two roller heads, each equipped with one‐way clutch, and is driven with one motor (Figure 2A). The one‐way clutch is a clutch that conveys a torsional force toward a single direction. The roller heads turn according to the direction of the rotational axis and the roller head that does not turn is referred to as the clamp. M‐CART is equipped with two multi‐ring‐type roller pump units (Figure 2B). Each roller pump is equipped with two roller heads arranged with the upper clockwise‐rotation roller head above the lower counterclockwise‐rotation roller head. When the rotational axis of the pump is rotated clockwise, only the upper roller head rotates, and when rotated counterclockwise, only the lower roller head rotates.

### 2.3 M‐CART

M‐CART is equipped with two multi‐ring‐type roller pump units (two motors), three pressure monitors, two electric clamps, one air detector, two filter holders, and two removable poles (Figure 2B). In M‐CART, 70% downsizing and 60% lightning of the weight were achieved by introducing two multi‐ring‐type roller pump units into the T‐CART, whose size was almost the same as that of the existing equipment (Figure 1).

**FIGURE 3:**
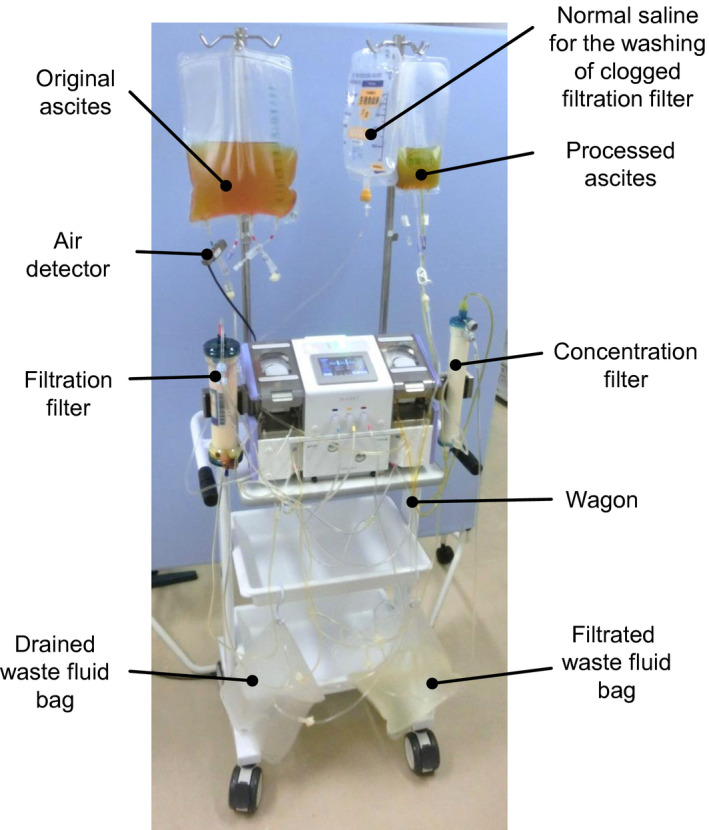
Representative photograph of ascites processing using M‐CART. M‐CART is compact, lightweight (25 kg), and easy to transport [Color figure can be viewed at wileyonlinelibrary.com]

**FIGURE 4:**
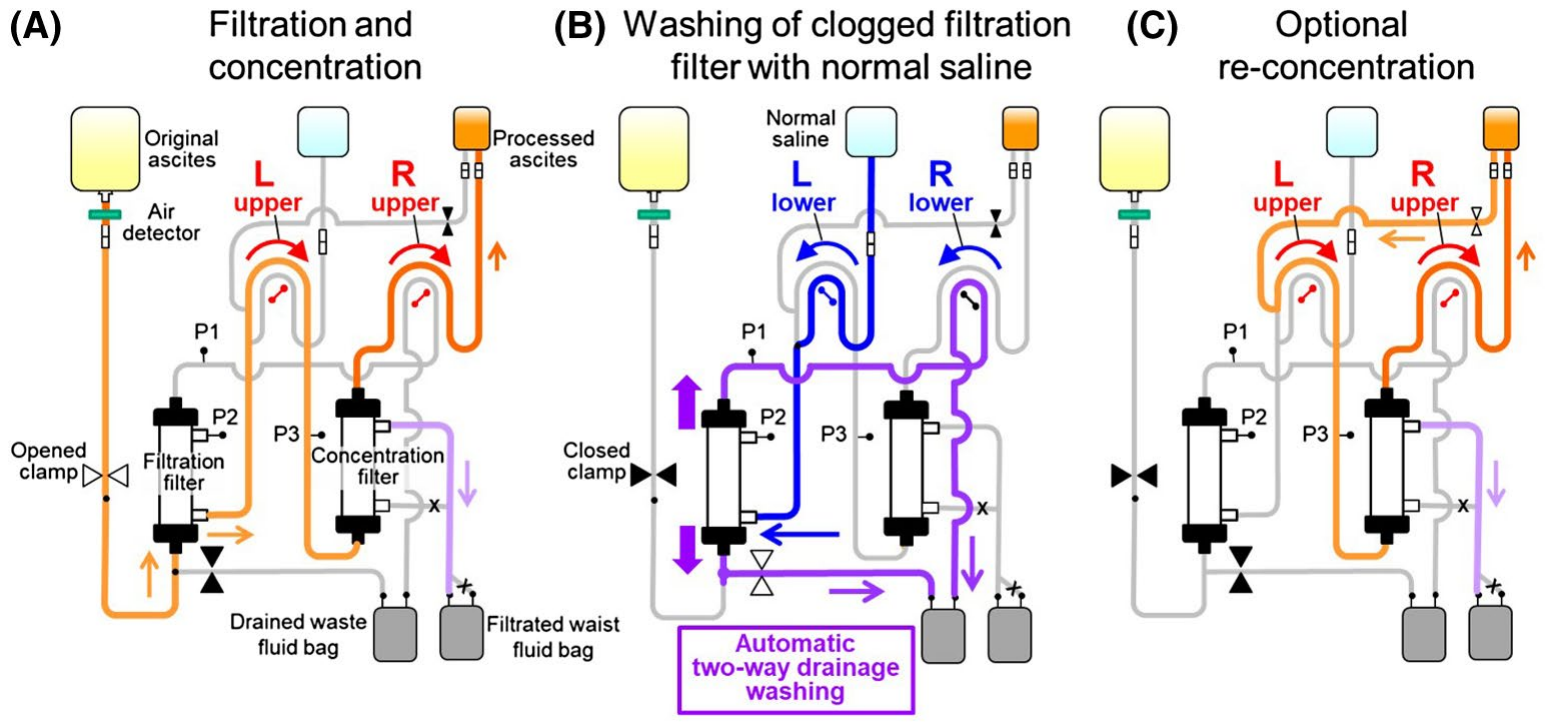
Circuit of M‐CART. The left‐side upper roller pump located between the filtration and concentration filters in the tube circuit produces negative pressure to drive the original ascites to the filtration filter and positive pressure to drive the filtrated ascites to the concentration filter. Ascites is filtrated from inside the hollow fibers to outside in the filtration filter (A). The filtration and concentration processing is temporarily discontinued by the rotational axis of two multi‐ring‐type roller pumps turning in the opposite direction when the TMP increased to higher than the set point by clogging of the filtration filter. The clogging substances in the filtration filter are removed from the lumen and pores of the hollow fibers, and header parts on both sides by infusing normal saline from the outside to the inside of the hollow fibers and draining the fluids in two directions (two‐way drainage washing) (B). Optional re‐concentration can be conducted when there is a large volume of processed ascites is processed (C). P1‐3, pressure monitor [Color figure can be viewed at wileyonlinelibrary.com]

Various automatic processes, including the response to clogging of the filtration and concentration filters (automatic washing of clogged filtration filter with normal saline and self‐regulation of the concentration ratio), were enabled (Table 1). Figures 3 and 4, respectively, present the photograph and circuit of M‐CART. The negative pressure driving force is produced by the left‐side upper pump located between the filtration and concentration filters in the tube circuit (Figure 4A). Ascites is filtrated from inside the hollow fibers to outside in the filtration filter. The left‐side upper pump also produces a positive pressure force to drive the filtrated ascites to the concentration filter. The left‐ and right‐side lower pumps are used for driving normal saline to the clogged filtration filter and for drainage of waste fluid from the upper header part of the filtration filter, respectively (Figure 4B).

**TABLE 1:** Structural equipment and automatic processing of developed CART equipment

				First model (T‐CART)	Compact model (M‐CART)
Structural equipment	Roller pump		Pump unit	3	2
			Roller head	3	4
			Motor	3	2
	Electric clamp			6	2
	Pressure monitor			6	3
	Touch panel			1	1
Automatic processing	I. Priming	1. Leak check and washing		**A**	**A**
	II. Filtration and concentration	2. Filtration and concentration		**A**	**A**
		3. Washing of clogged filtration filter with normal saline		**A**	**A**
		4. Self‐regulation of the concentration ratio		**A**	**A**
		5. Re‐concentration^a^		Not	**A**
	III. Finishing	6. Collection of the ascites in the circuit and filters		**A**	**A**
Type of tube circuit set for CART equipment				Panel type	Tube holder‐type

Abbreviations: A, available; Not, not available.

^a^ Optional function that can be selected by the operator.

Various safety measures such as alarm systems, support information systems, safety structures, and safety functions are implemented in M‐CART (Table 2). With the leak check function, the leak of the hollow fiber can be checked with high accuracy. M‐CART can detect human errors such as the incomplete connection of pressure lines, improper mounting of the roller pump tubes, improper closing of the two pump covers or the clamp cover, and incorrect opening/closing of the manual tube clamps. In order to completely attach the tube to the electric clamp, a new mechanism (clamp cover) was introduced in which the tube was attached to the groove of the clamp cover and the tube was attached to the clamp by closing the cover (Figure 2B). A snap‐in type filter holder was newly developed instead of the conventional clip type to reduce the risk of mixing‐up between the filtration and concentration filters with the same shape of tube joints (Figure 2B). This new type of filter holder takes advantage of the fact that only one type of filtration filter (AHF‐MO, Asahi Kasei Medical Co. Ltd., Tokyo, Japan) and one type of concentration filter (AHF‐UP, Asahi Kasei Medical Co. Ltd.) are sold, and the outer diameters of these two columns are different (AHF‐MO, 55 mm; AHF‐UP, 38 mm).

**TABLE 2:** Safety measures of the CART systems

Safety measure		CART system			
Alarm system: 1‐4 Support information system: 5 Safety structure and safety function: 6	Process	M‐CART	Conventional equipment with the mode for CART	Other method	
1. Detection of equipment error					
(1) System error of the equipment	I	**A**	**A**	**A**	Not
2. Detection of filter error					
(2) Leak of filtration filter	I	**A**	**A**	Not^c^	
3. Detection of a tube circuit setting error					
(3) Incomplete connection of the pressure line	I	**A**	Not	Not	
(4) Incomplete mounting of the pump tubing	I	**A**	Not	Not	
(5) Cover open (clamp cover/pump cover)	I/II/III	**A**	**A**	**A**	Not
4. Detection of abnormal processing status					
(6) Tube clamp^a^ (open/close)	I/II/III	**A**	**A**	**A**	Not
(7) Abnormal pump rotation	I/II/III	**A**	Not	Not	
(8) Abnormal pressure (P1/P2/P3/TMP)	I/II/III	**A**	**A**	**A**	Not
5. Support information					
(9) Bag empty (original ascites)	II	**A**	**A**	**A**	Not
(10) Bag exchange (processed ascites)	II	**A**	Not	Not	
(11) Bag exchange (drained waste fluid/filtrated waste fluid)	II	**A**	Not	Not	
(12) Preparation for clogged filtration filter washing	II	**A**	Not	Not	
(13) Achieve target process	I/II/III	**A**	**A**	Not	
6. Safety structure and safety function					
(14) Touch panel: for procedure guidance	I/II/III	**A**	**A**	**A**	Not
(15) Snap‐in type filter holder: to prevent mixed‐up incident	I	**A**	Not	Not	
(16) Clamp cover: to prevent incomplete tubing attachment	I	**A**	Not	Not	
(17) Pump cover lock structure: to prevent incidental opening	I/II/III	**A**	Not	Not	
(18) Tube holder: to keep connecting tips clean	I	**A**	Not	Not	
(19) Pump tube guide: to set pump tubes accurately	I	**A**	Not	Not	
(20) Closed‐circuit tubing set: to prevent contamination	I/II/III	**A**	**A**	Not	
(21) Automatic washing^b^ of clogged filtration filter and self‐regulation of the concentration ratio	II/III	**A**	Not	Not	

Abbreviations: A, available; I priming; II, filtration and concentration; III, finishing (collection of the ascites in the circuit and filters); Not, not available; P1, pre‐filtration filter pressure; P2, post‐filtration filter pressure; P3, pre‐concentration filter pressure; TMP, transmembrane pressure of the filtration filter.

^a^ Original ascites line, re‐concentration line, or normal saline line.

^b^ Two‐way drainage washing.

^c^ Performed manually.

Priming and ascites processing can be performed safely by monitoring the transmembrane pressure (TMP) of the filtration filter and entrance pressure of the concentration filter (Figure 4). The reference pressure value of the filtration and concentration filters is 500 mm Hg. An excessive increase in TMP in filters will cause breakage of hollow fibers. In M‐CART, automatic washing of clogged filtration filter is performed to prevent the breakage of the hollow fibers of the filtration filter or hemolysis of red blood cells in bloody ascites. The filtration and concentration process was temporarily discontinued when the TMP increased to higher than the setpoint by clogging of the filtration filter. The two multi‐ring‐type roller pumps were turned to the opposite direction and washing of the filtration filter with normal saline was performed automatically. Clogging of the header part on the entrance side was resolved by draining the fluids in two directions, the entrance side and opposite side, while washing the filtration filter (Two‐way drainage washing). Self‐regulation of the concentration ratio is also performed to prevent the breakage of the hollow fibers of the concentration filter and to concentrate ascites at a concentration ratio as close as possible to the set value. The concentration ratio reduces automatically when the entrance pressure of the concentration filter increases to higher than the upper limit of the setpoint by clogging of the concentration filter and the concentration ratio increases automatically when the entrance pressure decreases below the lower limit of the setpoint. Re‐concentration can optionally be conducted when there is a large volume of processed ascites (Figure 4C).

### 2.4 Tube holder‐type circuit set for M‐CART

**FIGURE 5:**
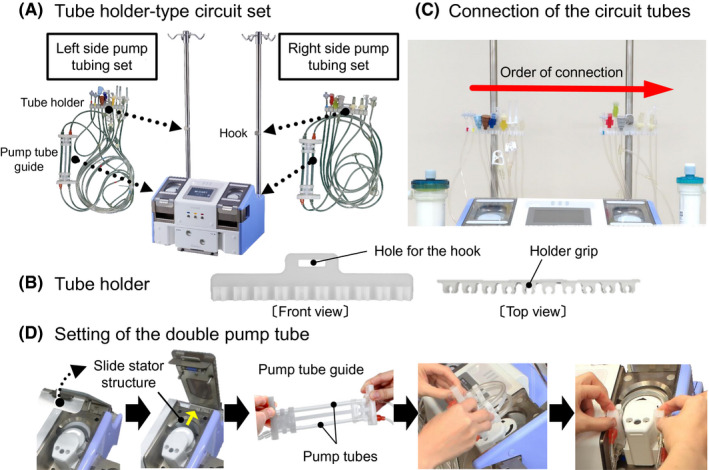
Setting of the tube holder‐type circuit set. Two tube circuits for the bilateral roller pump were stored in separate bags (A). The tips of the tubes were lined up in order of connection from the left side to the right side in the two tube holders (B) and hung on the hooks in two poles (C). The slide stator structure, which moves the pusher block back and forth when the pump cover opens and closes, and pump tube guide aid in the quick and accurate setting of the double pump tubes to the multi‐ring‐type roller pump unit (D) [Color figure can be viewed at wileyonlinelibrary.com]

We developed a tube holder‐type circuit set for M‐CART based on human engineering (Figure 5). Two tube circuits for the bilateral roller pump were stored in separate bags (Figure 5A). Two tube holders were hung on the hooks in two poles at the beginning of the circuit setting. Tips of tubes were lined up in order of connection from the left side to the right side in the two tube holders (Figure 5B,C). We developed a pump tube guide attached to a circuit tube and loaded M‐CART with a slide stator structure where a pusher block can be moved by opening and shutting off the pump cover so that a double tube could be attached to a roller pump easily and accurately (Figure 5D).

### 2.5 Usability evaluation of the priming process

**FIGURE 6:**
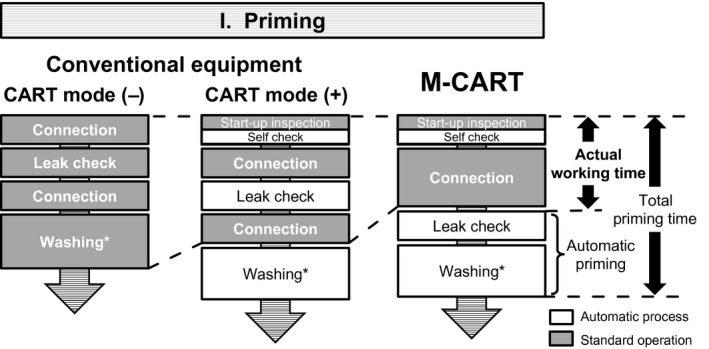
Comparison of the priming process. In M‐CART, a circuit set‐wearing process is performed at the beginning of the priming process, and a subsequent leak check and washing are automatically performed. Therefore, the actual working time has been reduced to less than half that of the conventional CART equipment without the mode for CART. *At the end of the step, replacement with heparinized normal saline can optionally be performed as an additional process

The usability of the priming process of M‐CART was evaluated by six clinical engineers with no experience using M‐CART. They had experienced using other types of the CART system more than 10 times. After watching a 15‐minute video orientation about the priming protocol and after receiving the priming protocol document, the participant worked on priming alone. The total priming time (the time from the equipment was switched on until circuit washing was accomplished) and actual working time (the amount of time the clinical engineer spent standing in front of M‐CART) were measured (Figure 6). The success rate (which refers to the completion of the priming process alone) was evaluated. Using a questionnaire, the satisfaction about (a) ease of use, (b) simplicity of the operation, (c) working time, (d) safety, (e) ease of learning (operation can be easily understood), (f) ease of mastering the procedure (operation is easy to memorize), (g) tube holder, and (h) pump tube guide was evaluated using a visual analog scale.^18^

### 2.6 Clinical evaluation

**FIGURE 7:**
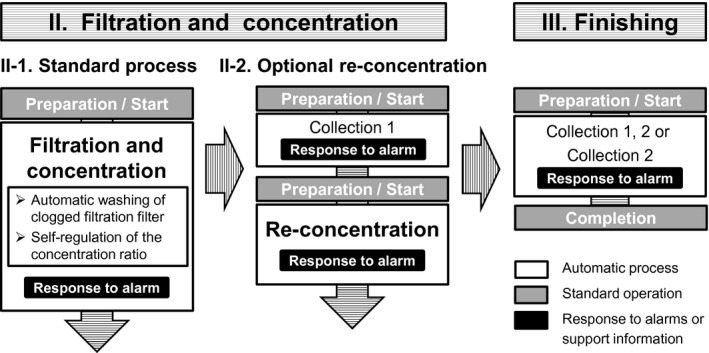
Ascites processing procedure in M‐CART. Re‐concentration (II‐2) is an optional process. Collection 1, ascites collection from the filtration filter; Collection 2, ascites collection from the concentration filter

At Tokushima University Hospital and five affiliated hospitals, 41 sessions of standard CART using M‐CART were performed in 17 patients with refractory ascites from September 2018 to April 2019. Filtration filter (AHF‐MO) and concentration filter (AHF‐UP) were used for ascites filtration and concentration, and the filtration flow rate was set at 50 mL/min. The ascites processing time (the time from the beginning of the connection of the original ascites bag to the tube circuit until the end of ascites processing), the standard operation time for the preparation of the following automatic process or the completion of ascites processing, the setting change or suspension time, and the response time to alarms and support information were calculated by analyzing the M‐CART operation logs (Figure 7). The automatic processing time was calculated by subtracting the standard operating time, the setting change or suspension time, and the response time to alarms or support information from the ascites processing time. A series of responses to the alarms and support information were counted as one response and the time from the beginning of the first response to the end of the last response was calculated as one response time. The initial preparation time (connection of the original ascites bag and input of set values), setting change time, and processing completion time (pressing the completion button) were added to the ascites processing time as 1 minute, 20 seconds, and 10 seconds, respectively. The condition of filtration and concentration (frequencies of automatic washing of clogged filtration filter and self‐regulation of the concentration ratio; the number of automatic washing of clogged filtration filter performed in one session), weight, and characteristic (specific gravity, protein concentration, albumin concentration, and immunoglobulin G [IgG] concentration) of the original and processed ascites were evaluated. The recovery rate was calculated as follows:

$$\begin{array}{c} \text{Recovery rate}\;\left(\%\right)\;=\;\\ \left(\text{processed ascitic concentration}\;\times \;\text{processed ascitic weight}\;/\;\text{processed ascitic specific gravity}\right)\\ /\left(\text{original ascitic concentration}\;\times \;\text{original ascitic weight}\;/\;\text{original ascitic specific gravity}\right)\;\times 100 \end{array}$$

Data about drugs used (anticoagulant, steroid, and antipyretics) and adverse events associated with the CART procedure were obtained. This study was approved by the Ethical Review Board of Tokushima University Hospital (3182‐1) and affiliated hospitals.

### 2.7 Statistical analysis

The data were expressed as mean ± standard deviation (SD). The Mann–Whitney *U* test and *χ* ^2^ test were used for the analysis of data. A *P* value of <0.05 was considered significant. Statistical analyses were performed by XLSTAT 2016 (Addinsoft Inc., NY, USA).

## 3 RESULTS

### 3.1 Usability evaluation of the priming process

**FIGURE 8:**
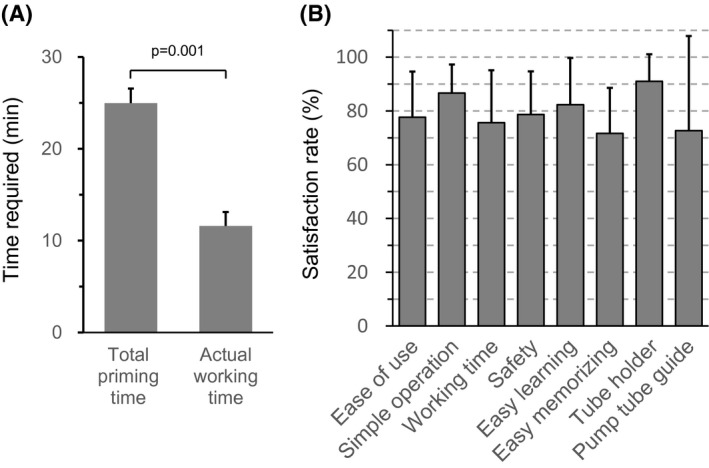
Priming time (A), and usability of the priming process and circuit set‐wearing aids (B). Values are expressed as mean ± standard deviation

The number of years that the participants worked as a clinical engineer and their years of experience performing blood purification duties such as hemodialysis were 12.3 ± 6.5 years and 10.8 ± 6.2 years, respectively. All the participants completed the priming process without any help. The actual working and priming times were 11.6 ± 1.5 minutes and 25.0 ± 1.6 minutes, respectively (Figure 8A). With regard to its usability, the average satisfaction rates of all the items were >70% (Figure 8B).

### 3.2 Clinical evaluation

Forty‐one sessions of standard CART were performed in 17 patients with refractory ascites using M‐CART (Table 3). Of them, nine had cancer (3, gastric cancer; 2, ovarian cancer; 1, ovarian granulosa cell tumor; 1, colorectal cancer; 1, bile duct cancer; and 1, gallbladder cancer) and eight had liver cirrhosis. Twenty‐two sessions of CART were performed in patients with malignant ascites and 19 sessions in those with hepatic ascites. The total amount of ascites was processed in all the sessions without any trouble. The weights of the collected and processed ascites were 4494 ± 2055 g and 560 ± 188 g, respectively, and the concentration ratio was 8.0 ± 2.5.

**TABLE 3:** Characteristics of ascites and automatic processing of M‐CART

Primary diagnosis	Number of sessions	Weight of original ascites (g)	Automatic processing (%)	
			Washing of clogged filtration filter	Self‐regulation of the concentration ratio
Malignancy	22 (53.7%)	3984 ± 1642 (1760‐6840)	50.0	90.9
Gastric cancer	10 (24.4%)	3126 ± 1490 (1760‐6840)	20.0	100.0
Ovarian granulosa cell tumor	6 (14.6%)	4858 ± 1262 (2910‐6160)	100.0	100.0
Ovarian cancer	2 (4.9%)	5820 ± 71 (5770‐5870)	100.0	100.0
Colon cancer	2 (4.9%)	3595 ± 2468 (1850‐5340)	50.0	100.0
Bile duct cancer	1 (2.4%)	2890	0	0
Gallbladder cancer	1 (2.4%)	5520	0	0
Liver cirrhosis	19 (46.3%)	5085 ± 2356 (1680‐11210)	10.5	10.5
Total	41	4494 ± 2055 (1680‐11210)	31.7	53.7

Note

Values are expressed as mean ± standard deviation (minimum–maximum).

**FIGURE 9:**
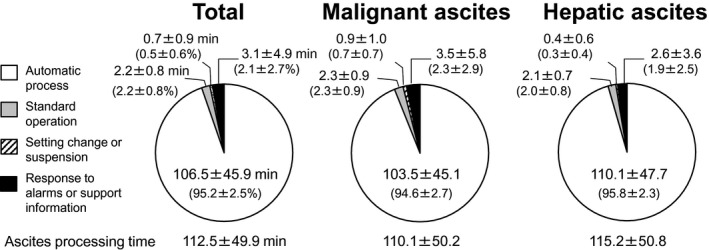
Ascites processing time of the total sessions, malignant ascites, and hepatic ascites. Approximately 95% of ascites processing was performed automatically. No significant differences were observed in the ascites processing time and rates of the standard operation and response to alarms or support information between the malignant and hepatic ascites. Values are expressed as mean ± standard deviation

The package insert of the filtration filter states that ascites treatment should be performed at a flow rate of ≤3000 mL/h to prevent fever.^4^, ^17^ For this reason, in this study, ascites processing was performed at a flow rate of 50 mL/min except for one case in which the processing speed was increased from 50 to 70 mL/min during the treatment of 9070 g of hepatic ascites. The mean ascites processing time was 112.5 ± 49.9 minutes (range: 48.6‐267.4 minutes) and a 106.5‐minutes (95.2%) ascites processing was performed automatically (Figure 9). The operator responded to alarms or support information at a mean of 3.2 ± 3.8 times (range: 0‐15 times; 3.1 ± 4.9 minutes, 2.1 ± 2.7% of ascites processing time). No significant difference was found between the malignant and hepatic ascites in the ascites processing time and the number of responses to alarms or support information. Human errors related to ascites processing were detected by M‐CART at an average of 0.4 times per session (2 times/41 sessions, improper closing of the pump or clamp cover; 17 times/41 sessions, incorrect opening/closing of manual tube clamps) and were appropriately addressed by the operator.

One washing of the clogged filtration filter took approximately 4 minutes and the number of washings and washing times per session were 0.5 ± 0.9 times (range: 0‐4 times) and 2.0 ± 3.7 minutes (range: 0‐16 minutes), respectively. The frequency of automatic washing of the filtration filter was 31.7%; the frequencies of automatic washing for malignant ascites and hepatic ascites were 50.0% and 10.5%, respectively (Table 3). The frequency of self‐regulation of concentration ratio was 53.7% and those for malignant and hepatic ascites were 90.9% and 10.5%, respectively. The frequency of re‐concentration was 14.6% (22.7%, malignant ascites; 5.3%, hepatic ascites).

The concentrations of protein, albumin, and IgG were significantly higher in malignant ascites than in hepatic ascites (Table 4). The recovery rates of protein, albumin, and IgG were 72.9 ± 10.9%, 72.9 ± 10.5%, and 71.2 ± 10.4%, respectively. The total doses of protein, albumin, and IgG were 65.9 ± 41.9 g, 34.9 ± 23.2 g, and 13.2 ± 9.4 g, respectively.

**TABLE 4:** Ascitic data and procedure of ascites processing of CART

	Total	Malignant ascites	Hepatic ascites	*P* value
Number of sessions	41	22 (53.7%)	19 (46.3%)	
Original ascites				
Weight of ascites (g)	4494 ± 2055 (1680‐11210)	3984 ± 1642 (1760‐6840)	5085 ± 2356 (1680‐11210)	0.191
Total protein (g/dL)	2.4 ± 1.5 (0.3‐4.6)	3.6 ± 0.9 (0.8‐4.6)	1.0 ± 0.7 (0.3‐2.4)	<.0001
Albumin (g/dL)	1.3 ± 0.8 (0.2‐2.6)	1.9 ± 0.5 (0.3‐2.6)	0.5 ± 0.3 (0.2‐1.2)	<.0001
IgG (mg/dL)	476 ± 309 (76‐1370)	656 ± 288 (254‐1370)	267 ± 172 (76‐680)	<.0001
Specific gravity	1.019 ± 0.008 (1.007‐1.033)	1.025 ± 0.005 (1.010‐1.033)	1.012 ± 0.004 (1.007‐1.020)	<.0001
Processed ascites				
Weight of ascites (g)	560 ± 188 (230‐890)	594 ± 207 (300‐890)	522 ± 161 (230‐880)	0.340
Total protein (g/dL)	12.3 ± 5.8 (2.3‐21.0)	16.5 ± 3.3 (5.3‐21.0)	7.4 ± 3.9 (2.3‐16.3)	<.0001
Albumin (g/dL)	6.5 ± 3.3 (1.3‐12.9)	8.9 ± 2.1 (2.2‐12.9)	3.8 ± 2.0 (1.3‐8.4)	<.0001
IgG (mg/dL)	2468 ± 1234 (538‐5890)	2988 ± 1182 (1150‐5890)	1865 ± 1020 (538‐3720)	0.005
Specific gravity	1.073 ± 0.031 (1.018‐1.120)	1.096 ± 0.017 (1.035‐1.120)	1.047 ± 0.022 (1.018‐1.092)	<.0001
Condition of ascites processing				
Concentration ratio	8.0 ± 2.5 (4.6‐18.4)	6.6 ± 1.1 (4.6‐9.3)	9.6 ± 2.8 (7.3‐18.4)	<.0001
Re‐concentration (%)	14.6	22.7	5.3	
Recovery rate				
Total protein (%)	72.9 ± 10.9 (46.8‐91.4)	68.7 ± 10.9 (46.8‐91.4)	77.9 ± 8.9 (60.7‐90.7)	0.009
Albumin (%)	72.9 ± 10.5 (49.9‐92.8)	70.3 ± 11.2 (49.9‐92.8)	75.9 ± 9.1 (61.1‐91.8)	0.126
IgG (%)	71.2 ± 10.4 (42.0‐89.1)	67.6 ± 11.3 (42.0‐87.5)	75.4 ± 7.5 (64.3‐89.1)	0.025
Recovery dose				
Total protein (g)	65.9 ± 41.9 (9.1‐159.3)	90.2 ± 39.0 (34.3‐159.3)	37.9 ± 24.2 (9.1‐86.6)	<.0001
Albumin (g)	34.9 ± 23.2 (5.0‐91.4)	48.5 ± 21.8 (14.2‐91.4)	19.3 ± 12.8 (5.0‐44.6)	<.0001
IgG (g)	13.2 ± 9.4 (1.9‐47.4)	16.6 ± 10.7 (4.1‐47.4)	9.3 ± 5.8 (1.9‐20.8)	0.013

Note

Values are expressed as mean ± standard deviation (minimum–maximum).

The options for the drugs (anticoagulants, steroids, and antipyretics) used during CART were determined by the treating physicians. As anticoagulant, low‐molecular‐weight heparin (2000 units/bag) was administered to the collected ascites at a frequency of 19.5%. Low‐molecular‐weight heparin was administered to all eight collected malignant ascites in one hospital and to seven of the nine malignant ascites in another hospital, and no anticoagulant was administered in the other four hospitals. Steroids were administered in 38 of the 41 sessions (92.7%) before processed ascites infusion to prevent fever (Table 5).

**TABLE 5:** Steroid premedication and frequency of fever

Primary diagnosis (number of sessions)	Premedication (mg)				BT	
	PSL	MPL	HYD	None	Fever	Increased BT (°C)
Malignancy (22)					5/22 (22.7%)	0.60 ± 0.67 (−0.4 to 2.3)
Gastric cancer (4)	30				1/4 (25.0%)	0.78 ± 1.04 (0‐2.3)
Gastric cancer (6)		20^a^			0/6	0.15 ± 0.44 (−0.4 to 0.9)
Ovarian granulosa cell tumor (6)	30				4/6 (66.7%)	1.00 ± 0.67 (0.1‐1.6)
Ovarian cancer (1)	30				0/1	1.0
Ovarian cancer (1)				None	0/1	0.7
Colon cancer (1)		20^a^			0/1	0.5
Colon cancer (1)			125		0/1	0.4
Bile duct cancer (1)		40			0/1	0.6
Gallbladder cancer (1)		20^a^			0/1	0
Liver cirrhosis (19)					2/19 (10.5%)	0.45 ± 0.46 (−0.4 to 1.7)
Liver cirrhosis (11)		40			1/11 (9.1%)	0.28 ± 0.39 (−0.4 to 1.2)
Liver cirrhosis (1)			500		0/1	0.9
Liver cirrhosis (5)			200		0/5	0.44 ± 0.21 (0.1‐0.6)
Liver cirrhosis (2)				None	1/2 (50.0%)	1.15 ± 0.78 (0.6‐1.7)
Total (41)					7/41 (17.1%)	0.53 ± 0.58 (−0.4 to 2.3)

Note

Fever was defined as a body temperature of ≥38.0°C and a 1°C increase from the pretreatment level. Values are expressed as mean ± standard deviation (minimum–maximum).

Abbreviations: BT, body temperature; HYD, hydrocortisone; MPL, methylprednisolone; PSL, prednisolone.

^a^ Combination with 50 mg of flurbiprofen axetil.

The body temperature increased by 0.53 ± 0.58°C (malignant ascites, 0.60 ± 0.67°C; hepatic ascites, 0.45 ± 0.46°C). Antipyretic was administered in four sessions (two sessions, acetaminophen 1000 mg; one session, acetaminophen 400 mg; one session, celecoxib 100 mg) and was effective. Adverse events occurred in 24.4% (10/41) of the sessions. A body temperature of ≥38.0°C and 1°C increase from the pre‐treatment level, which is the definition of fever by the Japan Society of Transfusion Medicine and Cell Therapy,^19^ were observed in 17.1% of the sessions (7/41; four sessions, ovarian granulosa cell tumor; one session, gastric cancer; two sessions, liver cirrhosis). Other adverse events included chest pain (1 session) during drainage of ascites; chills (2 sessions) and dyspnea (1 session) during reinfusion; and dyspnea and palpitation (1 session) after reinfusion. However, no other adverse events required clinical response or no severe adverse events were observed, and the patient fully recovered from the events at least by the next morning.

## 4 DISCUSSION

We built a consortium for medical‐engineering collaboration and developed a compact and automatic CART specialized equipment (M‐CART) using newly developed multi‐ring‐type roller pump units. M‐CART was approved under the Japanese regulations in 2018.

**FIGURE 10:**
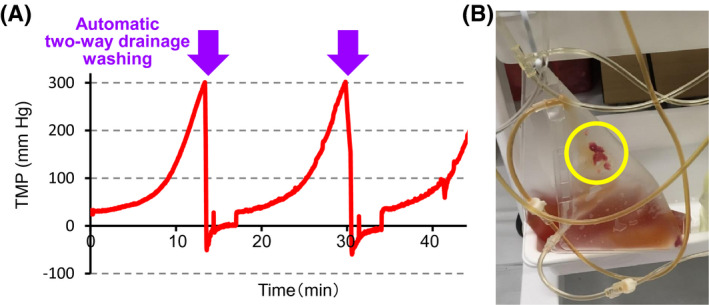
Representative transition of the TMP of the filtration filter in the case of filter washing (A) and the aggregate (circled part) of the header part on the entrance side of the filtration filter, which was drained into the waste fluid bag by two‐way drainage washing (B). M‐CART can prevent TMPs higher than the set value from being applied to the hollow fibers of the filtration filter by the automatic two‐way drainage washing [Color figure can be viewed at wileyonlinelibrary.com]

The M‐CART has some advantages compared with other equipment for CART. It primarily allows the processing of a large quantity of ascites without the constant assistance of an operator. When using an equipment without the mode for CART, the constant assistance of an operator for ascites processing was required. However, in M‐CART, the operation time by the operator was shortened by <5% of the ascites processing time, and the ascites processing of the total dose was possible in all the cases. This shortening of the working time was enabled by automatic processing functions especially automatic washing of clogged filtration filter with normal saline and self‐regulation of the concentration ratio. The response to filtration filter clogging in the conventional multi‐purpose blood processing equipment with the mode for CART was the reduction of filtration flow rate or drainage of clogging substances on the side opposite to the ascites entrance side. In some cases, the entire amount of ascites could not be processed.^6^, ^15^ However, in M‐CART, removal of the clogging substances from the lumen, pores of the hollow fibers, and header parts on both sides was performed by infusing normal saline from the outside to the inside of the hollow fibers and draining the fluids in two directions (Figure 4B and 10). Such powerful washing and self‐regulation functions of the concentration ratio had made the processing of the entire amount of ascites possible in all cases in our clinical evaluation. In particular, even in malignant ascites with high viscosity due to ovarian tumor, the entire amount of ascites could be treated by repeatedly washing the clogged filtration filter and self‐regulation of the concentration ratio. Since the main target of CART is changing from hepatic ascites to malignant ascites, these measures for filter clogging will become more important.

As a second advantage, various safety measures such as alarm systems, support information systems, safety structures, and safety functions are implemented in M‐CART (Table 2). The most important thing in safety management during ascites processing is to prevent harmful substances such as bacteria and cancer cells from being mixed into the processed ascites due to the breakage of the hollow fiber in the filtration filter. Washing a clogged filtration filter using a syringe carries a risk of damage to the hollow fiber by applying pressure higher than the tolerable pressure of 500 mm Hg in order to wash out the clogging substances by applying sufficient pressure. M‐CART can prevent TMPs higher than the set value from being applied to the hollow fibers of the filtration or concentration filters from the start of priming to the end of ascites processing. Snap‐in type filter holder, pump tube guide, and clamp cover, respectively, prevent incidents of mixing up between the filtration and concentration filters, incorrect pump tube installation, and incomplete mounting of clamp tubes. In addition, human errors such as the incomplete connection of the pressure lines, opening of the pump or clamp cover, and forgetting to close or open the manual clamps are detected. Warnings are given by sound and light, and the countermeasures are displayed on the touch panel.

As a third advantage, the M‐CART is compact and lightweight. Therefore, the operator can transfer the M‐CART to any area and can treat ascites simultaneously, while performing another work. It is also possible to treat ascites at the bedside. The multi‐ring‐type roller pump unit, which is equipped with a one‐way clutch, is a newly developed pump unit. The number of motors and electric clamps can be reduced through this pump unit. Equipped with two multi‐ring‐type roller pump units, it became possible to perform all automatic processes with two motors and two electric clamps.

Another advantage is that the priming operation of M‐CART is simple and easy. In M‐CART, the procedure from priming to completion of processing is displayed on a touch panel. Additionally, a circuit set wearing process is performed at the beginning of the priming process, and subsequent leak check and washing are automatically performed (Figure 6). Therefore, the operator can leave M‐CART in the second half of the priming process (leak check and washing the circuit) and the actual working time has been reduced to less than half that of the conventional equipment without the mode for CART. Based on the results of the survey, the satisfaction rate on the use of tube holder was the highest, accounting for 91.0%. The high satisfaction rate on the use of this tube holder was because the location of the connection tips and the connecting order were easy to understand, and the risk of the connecting tips falling on the floor and becoming unclean was reduced.

The efficiency of CART for processing ascites is usually evaluated based on the recovery rate and dose of protein. Table 6 shows the past report of recovery rate and dose of protein and albumin. Based on our clinical evaluation of M‐CART, an average of 4494 g of ascites was processed and the total amount of ascites was processed in all CART sessions. The protein, albumin, and IgG recovery rates (dose) were 72.9%, 72.9%, and 71.2% (65.9 g, 34.9 g, and 13.2 g), respectively. These values were equivalent to or greater than the reported levels.^2^, ^6^, ^7^, ^8^, ^9^, ^10^, ^13^, ^14^, ^15^, ^16^, ^20^, ^21^, ^22^, ^23^, ^24^, ^25^ The protein recovery rate decreases due to the following causes: (a) discontinuation of ascites processing due to clogging of the filtration or concentration filters; (b) loss of remaining ascites in the filters and circuit after ascites processing is completed; (c) loss of ascites due to washing of the filtration filter; and (d) protein adsorption to filter membrane. By M‐CART, the total amount of ascites can be processed by automatic washing of clogged filtration filter and self‐regulation of the concentration ratio. Furthermore, M‐CART is equipped with an ascites collection function at the end of ascites processing to improve the recovery rate (Figure 7). When the TMP of the filtration filter exceeded 300 mm Hg, automatic washing with 900 mL of normal saline was performed in our clinical evaluations. The average number of automatic washings with normal saline performed in one session was 0.5 times (0.9 times, malignant ascites; 0.1 times, hepatic ascites). Washing of the filtration filter with normal saline decreases the recovery rate as the ascites fluid in the filter is discarded. However, if the processing is discontinued without washing, the recovery rate will also decrease. Therefore, optimization of the timing of filtration filter washing and the washing technique used should be considered in further studies.

**TABLE 6:** Recovery rate and dose of protein and albumin by CART processing

Report	Equipment	Direction of filtration	Ascites (session)			Original ascites		Total amount of processing	Recovery rate (%)		Recovery dose (g)	
			Ca	LC	Other	Quantity	TP (g/dL)		TP	Alb	TP	Alb
1. JCSG et al.^13^	KM‐CART*	Out	11			2840 ± 660 mL	–	–	–	71.1 ± 9.6	–	–
2. Takahashi et al.^14^	P	In			6^a^	2533 ± 1039 mL	–	–	–	66.6 ± 9.4^b^	–	45.0 ± 30.1
3. Ito et al.^2^	P	In	81			2635 ± 1360 mL	4.2 ± 1.1	–	–	–	63.1 ± 39.4	–
4. Maeda et al.^20^	–	–	51			4007 ± 1304 mL	–	–	63.1 ± 14.9	63.4 ± 22.2	75.0 ± 29.8	39.3 ± 20.8
5. Ito et al.^7^	P	In	100			3197 ± 1424 mL	4.6 ± 1.1	–	–	–	93.1 ± 51.6	–
6. Wang et al.^21^	–	–	58			7730 ± 3390 mL	2.5 ± 1.0	–	–	–	161.2 ± 89.1	–
7. Yamaguchi et al.^10^	P	In	127			3056 ± 1250 mL	4.3 ± 0.8	–	–	–	85.5 ± 46.9	–
8. Maeda et al.^22^	KM‐CART	Out	46^c^			4900 ± 2100 mL	2.8 ± 1.3	–	51.6 ± 20.8	55.0 ± 20.6	58.8 ± 30.4	31.6 ± 17.5
	KM‐CART	Out	6^c^	6100 ± 2700 mL	4.2 ± 0.5	–	40.4 ± 16.9	44.6 ± 20.1	95.4 ± 45.5	58.1 ± 37.5
9. Hanafusa et al.^6^	P78.2%; D21.8%	In89.3%; Out10.7%	128^c^	17^c^	4^c^	3709 ± 1730 g	2.7 ± 1.5	91%	72.0 ± 18.1	73.8 ± 16.9	67.8 ± 41.6	37.8 ± 24.7
	P78.4%; D21.6%	In89.2%; Out10.8%				3498 ± 1590 g	2.9 ± 1.6	–	69.0 ± 19.3	–	–	–
10. Yamada et al.^16^	DC‐CART	Out	59^d^			4900^e^ g	2.5^e^	100%	62^e^	–	73^e^	–
11. Kawata et al.^23^	P	–	47			2937 ± 820 mL	5.3 ± 1.2	–	58.2 ± 23.3	–	85.0 ± 33.2	–
12. Yoshizawa et al.^15^	P	Out	4	18		4130 ± 1680 mL	–	90.9%	–	–	49.4 ± 18.0	–
13. Ito et al.^24^	P	In	43^c^			3207 ± 1427 mL	4.5 ± 1.2	–	–	–	91.3 ± 53.0	–
14. Iwasa et al.^8^	–	–		81		4727 ± 2207 g	1.2 ± 0.7	–	–	–	40.6 ± 22.1	18.4 ± 9.7
15. Ohashi et al.^25^	–	–	21			3235 ± 1338 mL	–	–	–	–	65.0 ± 48.6	31.0 ± 22.5
16. Yamada et al.^9^	DC‐CART	Out	79	16	13	4159 ± 2570 mL	–	–	59 ± 23	–	67.7 ± 51.2	36.4 ± 27.6
17. Our case	M‐CART	In	22	19		4494 ± 2055 g	2.4 ± 1.5	100%	72.9 ± 10.9	72.9 ± 10.5	65.9 ± 41.9	34.9 ± 23.2
	M‐CART	In	22			3984 ± 1642 g	3.6 ± 0.9	100%	68.7 ± 10.9	70.3 ± 11.2	90.2 ± 39.0	48.5 ± 21.8
	M‐CART	In		19		5085 ± 2356 g	1.0 ± 0.7	100%	77.9 ± 8.9	75.9 ± 9.1	37.9 ± 24.2	19.3 ± 12.8

Note

Values are expressed as mean ± standard deviation.

Abbreviations: Alb, albumin; Ca, cancer; D, drop type; DC‐CART, drop type with adjustable concentrator‐cell free and concentrated ascites reinfusion therapy; In, inside‐out; KM‐CART*, aspiration type without manual washing of clogged filtration filter; KM‐CART, aspiration type with manual washing of clogged filtration filter; LC, liver cirrhosis; Out, outside‐in; P, pump type; TP, total protein.

^a^ Sinusoidal obstruction syndrome.

^b^ *n* = 5.

^c^ Patient number.

^d^ Mostly malignant ascites.

^e^ Median.

Generally, the most problematic adverse event in CART is fever. The degree of fever is thought to be affected by the patient’s original disease and condition, ascitic cytokine concentration, prophylactic administration of antipyretic drugs, and ascites processing speed. In the CART in which positive pressure‐type filtration using a drop‐type method or a roller pump equipment was performed, fever appeared in 10.5% of the sessions.^6^ In the CART in which positive pressure‐type filtration from the outside of the hollow fibers to the inside using a roller pump equipment was conducted at a flow rate of 100 mL/min, steroid was administered in all sessions prophylactically, and the elevation of body temperature was 0.5 ± 0.6°C.^15^ In the DC‐CART in which drop‐type filtration from the outside of the hollow fibers to the inside was performed, a steroid was administered in 56% of the sessions prophylactically, and elevation of body temperature to >38°C appeared in 16.4% of the sessions.^16^ The negative pressure‐type filtration method using a roller pump was located after a filtration filter was adapted in M‐CART to prevent the risk of the cell components of the ascites producing causative agents of the fever by the mechanical stimulation of the roller pump. In this study, steroids were administered prophylactically in 92.7% of the sessions, ascites was processed at a flow rate of 50 mL/min, and fever occurred in 17.1% of the CART sessions and 23.5% of the patients (4/17) and the degree of increase in body temperature was 0.53 ± 0.58°C. However, the increase in body temperature disappeared at least in the next morning. Fever and other side reactions that consisted of the problem clinically were not found and the CART using M‐CART was thought to be safe similarly to the conventional CART methods. Further studies about the mechanism of fever in CART and the optimization of the ascites processing and steroid administration methods for fever prevention will be necessary.

M‐CART is an equipment approved under the Japanese regulations with various safety functions. The Japanese Society for Apheresis devised a safety standard for CART in 2018.^26^ In this safety standard, it was indicated that the medical equipment approved under the Japanese regulations should be used in accordance with the package insert and the use of uncertified equipment such as aspirator or syringe is not recommended; the use of specialized equipment with safety functions is recommended following the pump‐type method. The circuit set for M‐CART is a closed‐circuit system as recommended in the safety standard of CART to avoid bacterial contamination and retrograde infection.

The current ascites processing method adopts the inside‐out filtration and negative pressure‐type method. Through further clinical studies and fundamental research, the optimization of the ascites processing method (inside‐out or outside‐in filtration; positive or negative pressure‐type; and flow rate) should be performed.

## 5 CONCLUSIONS

We developed a novel cell‐free and concentrated ascites reinfusion therapy specialized equipment (M‐CART) with newly developed multi‐ring‐type roller pump units through collaboration between medicine and industry. M‐CART is a compact and lightweight CART specialized equipment approved under the Japanese regulations. This equipment has various safety measures and automatic processing functions such as automatic washing of clogged filtration filter using normal saline and self‐regulation of the concentration ratio, so that M‐CART can safely and easily process a large quantity of ascites without the constant assistance of an operator.

## CONFLICT OF INTEREST

TO, MF, and T. Takayama served as a speaker or chair of the research lecture supported by the Takatori Corporation and received honoraria. TO, MS, and T. Takayama received research funding from Takatori Corporation. TO, MS, TN, YK, KK, YT, TM and HA are the inventors of the issued or pending patents owned by Tokushima University and/or Takatori Corporation. KK, YT, TM, YD, HA, and HF are the employees of Takatori Corporation. The remaining authors have no conflicts of interest to declare. The development of the T‐CART, M‐CART, and circuit set was supported by grants from METI (Grant No. 25‐017), AMED (Grant No. 25‐017), and NEDO (Grant No. 27J1130). This development was also supported by joint research fund from Takatori Corporation to Tokushima University. The usability evaluation of the priming process and the clinical evaluation were conducted under a contract for joint research between Takatori Corporation and Tokushima University and supported by joint research fund from Takatori Corporation to Tokushima University. For clinical evaluation, Takatori Corporation lent equipments (M‐CART) to affiliated hospitals. The data of the usability evaluation of the priming process and clinical evaluation were analyzed only by the members of Tokushima University.

## AUTHOR CONTRIBUTIONS

*Development of M‐CART:* TO, MS, TN, AT, YK, HY, KK, YT, TM, YD, HA, HF, and T. Takayama

*Development of circuit set:* TO, MS, TN, HY, TK, YO, MF, KK, YT, TM, YD, HA, HF, and T. Takayama

*Concept/design of evaluation:* TO, MS, TN, HY, TK, and T. Takayama

*Data analysis/interpretation:* TO, MS, TN, JN, NI and T. Takayama

*Drafting article:* TO and MS

*Critical revision of article:* TO, MS, TN, and T. Takayama

*Approval of the study:* TO, MS, TN, KT, T. Tomonari, T. Taniguchi, AT, YK, JN, NI, HY, TK, YO, MF, MI, H. Shibata, H. Shinomiya, MN, FK, and T. Takayama

*Statistical analysis:* TO and MS

*Funding secured:* KK, YT, TM, YD, HA, and HF

*Data collection:* KT, T. Tomonari, T. Taniguchi, TK, YO, MF, MI, H. Shibata, H. Shinomiya, MN, FK, and T. Takayama

## References

[1] Inoue N , Yamazaki Z , Oda T , Sugiura M , Wada T . Treatment of intractable ascites by continuous reinfusion of the sterilized, cell‐free and concentrated ascitic fluid. Trans Am Soc Artif Intern Organs. 1977;23:699–702. 910402

[2] Ito T , Hanafusa N , Fukui M , Yamamoto H , Watanabe Y , Noiri E , et al. Single center experience of cell‐free and concentrated ascites reinfusion therapy in malignancy related ascites. Ther Apher Dial. 2014;18:87–92. 24499089 10.1111/1744-9987.12049

[3] Ueda T , Maehara M , Takahashi Y , Nakayama N , Kondo H , Shirota K , et al. Clinical significance of cell‐free and concentrated ascites re‐infusion therapy for advanced and recurrent gynecological cancer. Anticancer Res. 2012;32:2353–7. 22641674

[4] Takamatsu S , Miyazaki H , Katayama K , Sando T , Takahashi Y , Dozaiku T , et al. The present state of cell‐free and concentrated ascites reinfusion therapy (CART) for refractory ascites: focusing on the clinical factors affecting fever as an adverse effect of CART (in Japanese). Kan Tan Sui. 2003;46:663–9.

[5] Kozaki K , IInuma M , Takagi T , Fukuda T , Sanpei T , Terunuma Y , et al. Cell‐free and concentrated ascites reinfusion therapy for decompensated liver cirrhosis. Ther Apher Dial. 2016;20:376–82. 27523078 10.1111/1744-9987.12469

[6] Hanafusa N , Isoai A , Ishihara T , Inoue T , Ishitani K , Utsugisawa T , et al. Safety and efficacy of cell‐free and concentrated ascites reinfusion therapy (CART) in refractory ascites: Post‐marketing surveillance results. PLoS ONE. 2017;12:e0177303. 28510606 10.1371/journal.pone.0177303 PMC5433707

[7] Ito T , Hanafusa N , Iwase S , Noiri E , Nangaku M , Nakagawa K , et al. Effects of cell‐free and concentrated ascites reinfusion therapy (CART) on symptom relief of malignancy‐related ascites. Int J Clin Oncol. 2015;20:623–8. 25239690 10.1007/s10147-014-0750-y

[8] Iwasa M , Ishihara T , Kato M , Isoai A , Kobayashi R , Torii N , et al. Cell‐free and concentrated ascites reinfusion therapy for refractory ascites in cirrhosis in post‐marketing surveillance and the role of tolvaptan. Int Med. 2019;58:3069–75. 10.2169/internalmedicine.3091-19 PMC6875447 31292400

[9] Yamada Y , Inui K , Hara Y , Fuji K , Sonoda K , Hashimoto K , et al. Verification of serum albumin elevating effect of cell‐free and concentrated ascites reinfusion therapy for ascites patients: a retrospective controlled cohort study. Sci Rep. 2019;9:10195. 31308465 10.1038/s41598-019-46774-9 PMC6629637

[10] Yamaguchi H , Kitayama J , Emoto S , Ishigami H , Ito T , Hanafusa N , et al. Cell‐free and concentrated ascites reinfusion therapy (CART) for management of massive malignant ascites in gastric cancer patients with peritoneal metastasis treated with intravenous and intraperitoneal paclitaxel with oral S‐1. Eur J Surg Oncol. 2015;41:875–80. 25986856 10.1016/j.ejso.2015.04.013

[11] Kimura Y , Harada Y , Yasuda N , Ishidao T , Yusa S , Matsusaki K , et al. Effective recovery of highly purified CD326 (+) tumor cells from lavage fluid of patients treated with a novel cell‐free and concentrated ascites reinfusion therapy (KM‐CART). SpringerPlus. 2015;4:780. 26702369 10.1186/s40064-015-1508-3 PMC4683161

[12] Katoh S , Kojima T , Itoh K , Yoneyama S , Ida K , Nakaji S . Usefulness of a nonmachinery based system for the reinfusion of cell‐free and concentrated autogenous ascitic fluid. Artif Organs. 1997;21:1232–8. 9423974 10.1111/j.1525-1594.1997.tb00483.x

[13] Japanese CART Study Group , Matsusaki K , Ohta K , Yoshizawa A , Gyoda Y . Novel cell‐free and concentrated ascites reinfusion therapy (KM‐CART) for refractory ascites associated with cancerous peritonitis: its effect and future perspectives. Int J Clin Oncol. 2011;16:395–400. 21347629 10.1007/s10147-011-0199-1

[14] Takahashi H , Sakai R , Fujita A , Kuwabara H , Hattori Y , Matsuura S , et al. Concentrated ascites reinfusion therapy for sinusoidal obstructive syndrome after hematopoietic stem cell transplantation. Artif Organs. 2013;37:932–6. 23692354 10.1111/aor.12080

[15] Yoshizawa M , Nakatsuji Y . Improvement of major problems in the cell‐free and concentrated ascites reinfusion therapy system—constructing of cell‐free and concentrated ascites reinfusion therapy system using external pressure for filtration. Ther Apher Dial. 2019;23:233–6. 31033153 10.1111/1744-9987.12829

[16] Yamada Y , Harada M , Yamaguchi A , Kobayashi Y , Chino T , Minowa T , et al. Technical performance and clinical effectiveness of drop type with adjustable concentrator‐cell free and concentrated ascites reinfusion therapy. Artif Organs. 2017;41:1135–44. 28589706 10.1111/aor.12933

[17] Package insert of ascites filtration filter AHF‐MP (in Japanese). Available from: http://www.asahi‐kasei.co.jp/medical/pdf/apheresis/ahf‐mo_document.pdf

[18] Flynn D , van Schaik P , van Wersch A . A comparison of multi‐item Likert and visual analogue scales for the assessment of transactionally defined coping function. Eur J Psychol Assess. 2004;20:49–58.

[19] The Japan Society of Transfusion Medicine and Cell Therapy. The Practical Guide for Management of Transfusion Reactions (Version 1.0). 2014 p. 41–2.

[20] Maeda O , Ando T , Ishiguro K , Watanabe O , Miyahara R , Nakamura M , et al. Safety of repeated cell‐free and concentrated ascites reinfusion therapy for malignant ascites from gastrointestinal cancer. Mol Clin Oncol. 2014;2:1103–6. 25279205 10.3892/mco.2014.335 PMC4179783

[21] Wang L , Okubo T , Shinsaka M , Kobayashi A , Ogasawara M , Sakaguchi R , et al. Efficacy and safety of cell‐free and concentrated ascites reinfusion therapy (CART) in gynecologic cancer patients with a large volume of ascites. J Obstet Gynaecol Res. 2015;41:1614–20. 26177394 10.1111/jog.12763

[22] Maeda S , Yabuuchi J , Nobuta H , Makiishi T , Hirose K . Characteristics of patients and their ascites who underwent repeated cell‐free and concentrated ascites reinfusion therapy. Ther Apher Dial. 2015;19:342–8. 26386222 10.1111/1744-9987.12343

[23] Kawata Y , Nagasaka K , Matsumoto Y , Oda K , Tanikawa M , Sone K , et al. Usefulness of cell‐free and concentrated ascites reinfusion therapy in the therapeutic management of advanced ovarian cancer patients with massive ascites. Int J Clin Oncol. 2019;24:420–7. 30474762 10.1007/s10147-018-1371-7

[24] Ito T , Hanafusa N , Iwase S , Noiri E , Nangaku M , Nakagawa K , et al. Ascitic IL‐10 concentration predicts prognosis of patients undergoing cell‐free and concentrated ascites reinfusion therapy. Ther Apher Dial. 2020;24:90–5. 31157953 10.1111/1744-9987.12863

[25] Ohashi A , Nakai S , Yamada S , Kato M , Hasegawa M. . A method for stabilizing the proportion of the reduced form of albumin during cell‐free and concentrated ascites reinfusion therapy in patients with malignant ascites. Ther Apher Dial. 2019;23:242–7. 31033167 10.1111/1744-9987.12827

[26] The Japanese Society for Apheresis, A safety standard of CART in 2018 (in Japanese). Available from: https://www.apheresis-jp.org/110790

